# Current Hospital Topics

**Published:** 1906-12-08

**Authors:** 


### HOSPITAL ADMINISTRATION.

### CONSTRUCTION AND ECONOMICS.

### CURRENT HOSPITAL TOPICS.

### Derbyshire Royal Infirmary.

Mr. Walter G. Carnt held the post of Secretary-Superintendent of this Infirmary for 14 years with such success that, on his appointment to a similar office at the Manchester Royal Infirmary, not only has the Board recorded upon their minutes the expression of their appreciation of his services to the Institution, but the Governors at the annual meeting, just held, passed a vote of thanks to Mr. Carnt, and the President and other speakers declared he had proved himself to be such a zealous and earnest worker that it would be no easy matter to replace him at Derby. The Board has just appointed Mr. Edmund Forster, M.A., Secretary of the Bradford Royal Infirmary, as Mr. Carnt's successor. The final selection was made from Mr. Forster, Mr. Herman Burney of the Royal Berkshire Hospital, and Mr. W. D. Hughes of the Swansea General and Eye Hospital. Mr. Forster was only appointed to Bradford from the Wolverhampton General Hospital in October last. Mr. Forster received his introduction to hospital work at the Royal London Ophthalmic Hospital under Mr. R. J. Bland.

### The Late George Herring.

At the same meeting of the Hospital Sunday Fund Council a resolution of sympathy and regret, and of appreciation of the generosity and services of Mr. George Herring was unanimously passed on the motion of Lord Stamford seconded by Sir William Church. The property left by Mr. Herring amounts to about £1,370,000, of which £600,000 will, it is expected, come to the Sunday Fund. That amount may be increased by £60,000, should the Salvation Army's "Back to the Land" scheme not prove successful. At Mr. Holland's suggestion a marble bust of Mr. Herring will be placed by the Sunday Fund in the Mansion House, with the consent of the Lord Mayor. The letter of appreciation which His Majesty King Edward VII. wrote to Mr. Herring will be engraved on the pedestal of this bust. We think that this bust, whilst it worthily and permanently records the munificence of the Fund's greatest benefactor, will prove of infinite value, as an object lesson, in years to come, and may be the means of leading other wealthy and generous city men to devote a portion of their wealth to the cause of the sick. No one who had an intimate knowledge of Mr. Herring can have failed to appreciate the straightforward directness of his character, or his disinterestedness and unselfish methods which must make his munificent charity fruitful as an example and an encouragement to the best type of philanthropists.

### Metropolitan Hospital Fund.

At the meeting of the Council of this Fund held on 29th ult. the origin of the fund in London was incidentally referred to in connection with a vote of condolence with Lady Waterlow on the death of Sir Sidney Waterlow, who since the inauguration of the fund had been one of its most prominent workers and warmest friends. The credit of introducing Hospital Sunday to London belongs primarily to the late Dr. James Wakley, the Editor of the *Lancet*, who repeatedly urged upon successive Lord Mayors and on the London clergy and ministers of religion the duty of organising a Metropolitan Hospital Sunday Fund. Dr. Wakley was untiring and unceasing in his advocacy of the movement for years. At length Sir Sidney Waterlow became Lord Mayor in 1872, when he took up the movement and organised the first Hospital Sunday collections in the metropolis in 1873. Sir Sidney Waterlow expressed the hope that he might live to see the total amount collected by the Fund raised to £1,000,000. That wish was gratified, and Canon Fleming's eloquent tribute to the continuous zeal and interest displayed by Sir Sidney Waterlow in this fund for 30 years, was well deserved and much appreciated by the Council. The London hospitals have had few better friends than Dr. James Wakley and Sir Sidney Waterlow. To them they owe, in large measure, their continued prosperity, and it is hoped that others will be found from time to time to carry on the work, and to make it even more successful in the future than it has been in the past.

